# Effect of multi-strain probiotics on the performance of AA+ male broilers

**DOI:** 10.3389/fvets.2022.1098807

**Published:** 2022-12-15

**Authors:** Qiangqiang Zou, Weishuang Meng, Tieliang Wang, Xiao Liu, Desheng Li

**Affiliations:** ^1^College of Animal Husbandry and Veterinary Medicine, Jinzhou Medical University, Jinzhou, China; ^2^College of Animal Science and Technology, Northeast Agricultural University, Harbin, China

**Keywords:** probiotic complex, AA+ broiler, growth performance, fecal microbiota, noxious gas

## Abstract

The aim of the experiment was to investigate the effects of a probiotic complex (PC) consisting of *Bacillus subtilis, Clostridium butyricum* and *Enterococcus faecalis* on productive performance, carcass traits, immune organ indices, fecal microbiota counts and noxious gas emissions in AA+ male broilers. Three hundred and sixty 1-day-old AA+ male broilers with similar body weight (44.77 ± 0.25) were randomly divided into 3 treatment groups of 6 replicates each, with 20 broilers in each replicate. The experimental groups consisted of a group fed a basal diet and groups fed basal diet supplemented with 0.1 and 0.2% PC. The results showed that the addition of PC had no significant effect (*P* > 0.05) on growth performance, and carcass traits of AA+ broilers during the experimental period (1–42 days of age). Dietary addition of PC significantly increased the thymus index of AA+ broilers (*P* < 0.05), reduced the number of *E. coli* and *Salmonella* in feces (*P* < 0.01) and reduced the concentrations of fecal NH_3_ and H_2_S emissions (*P* < 0.01). Furthermore, birds fed 0.2% PC diet had the highest number of fecal *Lactobacillus* counts. Results indicate that probiotic complex consisting of *Bacillus subtilis, Clostridium butyricum* and *Enterococcus faecalis* enhances immune organ development, reduces the number of *E. coli* and *Salmonella* in feces, increases the number of *Lactobacillus* and reduces NH_3_ and H_2_S emissions in feces. This trial provides a theoretical basis for the use of probiotic complexes in broiler production.

## Introduction

Since 2006, many countries had imposed strict restrictions or bans on the use of antibiotics in livestock and poultry (1). Therefore, the search for an environmentally friendly feed additive that can replace antibiotics became a hotspot for animal nutrition researchers. Probiotics was defined as “live microorganisms that, when given in sufficient quantities, are capable of providing health benefits to the host” (2). Many studies had demonstrated that probiotics can consistently induce positive effects on gastrointestinal tract morphology, microbiota, nutrient absorption, and immune response (3–6).

*Bacillus subtilis* was a widely used probiotic with various positive effects such as regulating intestinal microecological balance, improving nutrient utilization, promoting animal growth and development, and improving body immunity (7, 8). It was reported that supplementation of *Bacillus subtilis PB6* in broiler diets improved overall broiler performance, with significant improvements in body weight, feed conversion ratio (FCR), fluff morphology and European efficiency factor (EEF) (9). Similarly, supplementation of *Bacillus subtilis B2A* in broiler increased productivity, showed increases in bursal weight and reduce in intestinal *Salmonella* counts (10). *Clostridium butyricum* was also a common probiotic and when metabolism occurs, the products include both short chain fatty acids (SCFA), antimicrobial substances, vitamins, and a variety of enzymes (11). The enzymes produced by *Clostridium butyricum* speed up the digestion and absorption of nutrients and improve the body's immunity (12). Work had shown that the addition of preparations containing *Clostridium butyricum* improved the growth performance of broilers, increasing average daily gain (ADG) and average daily feed intake (ADFI) from 1 to 42 days of age (13). The addition of *Clostridium butyricum* significantly increased the ADG of broilers from 1–21 days of age (14). *Enterococcus faecalis*, which had the advantage of colonization over other probiotics, is also used more widely in pharmaceuticals and microbial additives. The addition of *Enterococcus faecalis* to the diet can soften the fiber in the feed, improved the utilization of the feed, promoted the growth of the animal organism and improved the meat quality of the livestock (15, 16).

Based on the probiotic properties of *Bacillus subtilis, Clostridium butyricum* and *Enterococcus faecalis*, the effects of adding them as a single strain to broiler diets had been reported by many authors. However, there are few reports on whether a combination of these three probiotics will have a beneficial effect on AA+ broiler diets. It was hypothesized that there were synergies among different probiotics, the combination of these three probiotics may receive more benefits than the single supplementation. Therefore, product combined *Bacillus subtilis, Clostridium butyricum* and *Enterococcus faecalis* were added into diets and investigate the effects of the probiotics complex on the productive performance, slaughter performance, intestinal morphology, fecal microbiota, and noxious gas emissions of AA+ broilers.

## Materials and methods

### Ethics statement

The Animal Conservation and Utilization Committee of the JZMU approved the animal use agreement (No. JZMULL2021006).

### Probiotic sources

The PC for this test was provided by Liaoning Kaiwei Biotechnology Co. The main components of PC were *Bacillus subtilis* (2 x 10^8^ cfu/g), *Clostridium butyricum* (2 x 10^6^ cfu/g) and *Enterococcus faecalis* (1 x 10^6^ cfu/g).

### Animals and experimental design

The trial was a completely randomized group design. Three hundred and sixty 1-day-old AA+ male broilers of similar weight (44.77 ± 0.25) and health were selected and randomly divided into 3 groups of 6 replicates each, with 20 broilers in each replicate. The experimental group consisted of a base diet group fed a basic basal diet and a base diet group fed 0.1 and 0.2% of PC.

### Animals feeding management

The 42-day trial was conducted at the AA+ Broiler Breeding Base from June 8, 2021 to July 19, 2021. The formulated (Table 1) were developed to meet the nutritional requirements recommended by the National Research Council (NRC, 1994). The birds had access to feed and water ad libitum. The broilers house was a fully enclosed house with an automatic environmental control system to ensure the optimal temperature and humidity. A total of 48 pens were provided for the test broilers to live in. Every two pens were a repeat. Every 10 birds were housed in a pen at a density of approximately 625 cm^2^ per broilers. The house temperature was 33°C for 1–3 d during the feeding period, and then decreased by 3°C per week to maintain a room temperature of about 22°C. The humidity was controlled at 40–70%. Throughout the experimental period, the 1–7 d and 36–42 d lighting programme was 24 h of light per day. The 8–30 d lighting program was provided 20 h per day with 4 h of darkness. The dark time was gradually reduced after 31 d.

**Table 1:** Composition and nutrient levels of the basal diet.

	**Contents**	
**Items**	**Days 1–21**	**Days 22–42**
**Ingredients (%)** ^**a**^	
Corn	60.4	64.05
Soybean meal	34.4	30
CaHPO_4_	1.40	1.30
CaCO_3_	1.21	1.12
NaCl	0.25	0.25
Soybean oil	1.00	2.00
Choline chloride	0.05	0.05
Lysine	0.08	0.10
*DL*-Met	0.21	0.13
Premix	1	1
Total	100.00	100.00
**Nutrient levels (%)** ^**b**^	
ME (MJ/Kg)	12.14	12.51
CP	21.17	19.24
Available phosphorus	0.38	0.36
Lys	1.29	1.15
Met	0.67	0.48
Met + Cys	1.00	0.72
Ca	0.92	0.87

a per kg of premix provides. VA, 5000 IU; VD, 10000 IU; VE, 75.0 IU; VK_3_, 18.8 mg; VB_1_, 9.8 mg; VB_2_, 28.8 mg; VB_6_, 19.6 mg; VB_12_, 0.1 mg; Biotin, 2.5 mg; Folic Acid, 4.9 mg; D-Pantothenic acid, 58.8 mg; Nicotinic acid, 196.0 mg; Zn, 37.6 mg; Fe, 40.0 mg; Cu, 4.0 mg; Mn, 50.0 mg; I, 0.2 mg; Se, 0.2 mg.

b The nutrient levels were calculated values.

### Test indicator determination

#### Growth performance

Birds were weighed on days 1, 21, and 42, and feed consumption was recorded on a pen basis throughout the experiment to calculate average daily gain (ADG), average daily feed intake (ADFI) and feed-to-weight ratio (F/G). After the broilers were sold at 42 days of age, the European Productivity Efficiency Factor (EPEF) was calculated. EPEF = [survival rate (%) x body weight (kg) x 10000]/ [age (d) x feed-to-meat ratio].

### Carcass traits and Immune organ index

At 42 days of experiment, one bird was randomly selected from each replicate and then fasting for 12 h. After weight of live weight, birds were killed by cervical dislocation carcass weight, leg muscle weight and pectoral muscle weight were determined and the slaughter rate, pectoral muscle rate, and leg muscle rate were calculated. The spleen, thymus and bursa were isolated, blotted with filter paper to remove blood stains and weighed fresh after removing surface fat and connective tissue, and the immune organ index was calculated.

Immune organ index = fresh weight of organ (g) / live weight before slaughter (kg)

### Fecal microbiota

Samples of 1 g of birds' feces were taken by replicates at 21 and 42 days of age and transported to the laboratory on ice according to the method of Dang et al. (17). The 1 g fecal sample from each replicate was diluted with 9 mL of 1% peptone broth and mixed. The viable counts of *E. coli, Lactobacillus* and *Salmonella* in the fecal samples were determined in a biosafety cabinet by measuring them on McConkey agar plates, MRS agar plates and BS agar plates respectively (in 10 g/L peptone solution). Microbial populations were finally expressed as log_10_ colony forming units per gram of feces.

### Fecal noxiousgas emissions

At 42 days of age, 150 g fresh bird manure was collected from each replicate and NH_3_ and H_2_S emissions from the manure were determined following the method of Dang et al. (17). Briefly, feces were placed in a 2 L plastic box, punch a small hole in the side of the box and seal it with tape. The boxes containing the feces were fermented at room temperature (25°C) for 6, 12, 24, and 48 h. The air sample is then collected with a gas collection pump from above the small hole in the side of the box. After each collection of air samples, reseal the box with tape. Concentrations of NH_3_ and H_2_S were measured in the range 0.00–100.00 mg/m^3^.

### Data analysis

The data was designed using a completely randomized grouping design. Replicate cage serves as the experimental unit. Multiple comparisons of significant differences in means were performed using the one-way ANOVA LSD method and visualization was completed using Graphpad Prism 8. Results were expressed as mean and standard error, with *P* < 0.05 indicating significant differences.

## Results

### Growth performance

**Figure 1:**
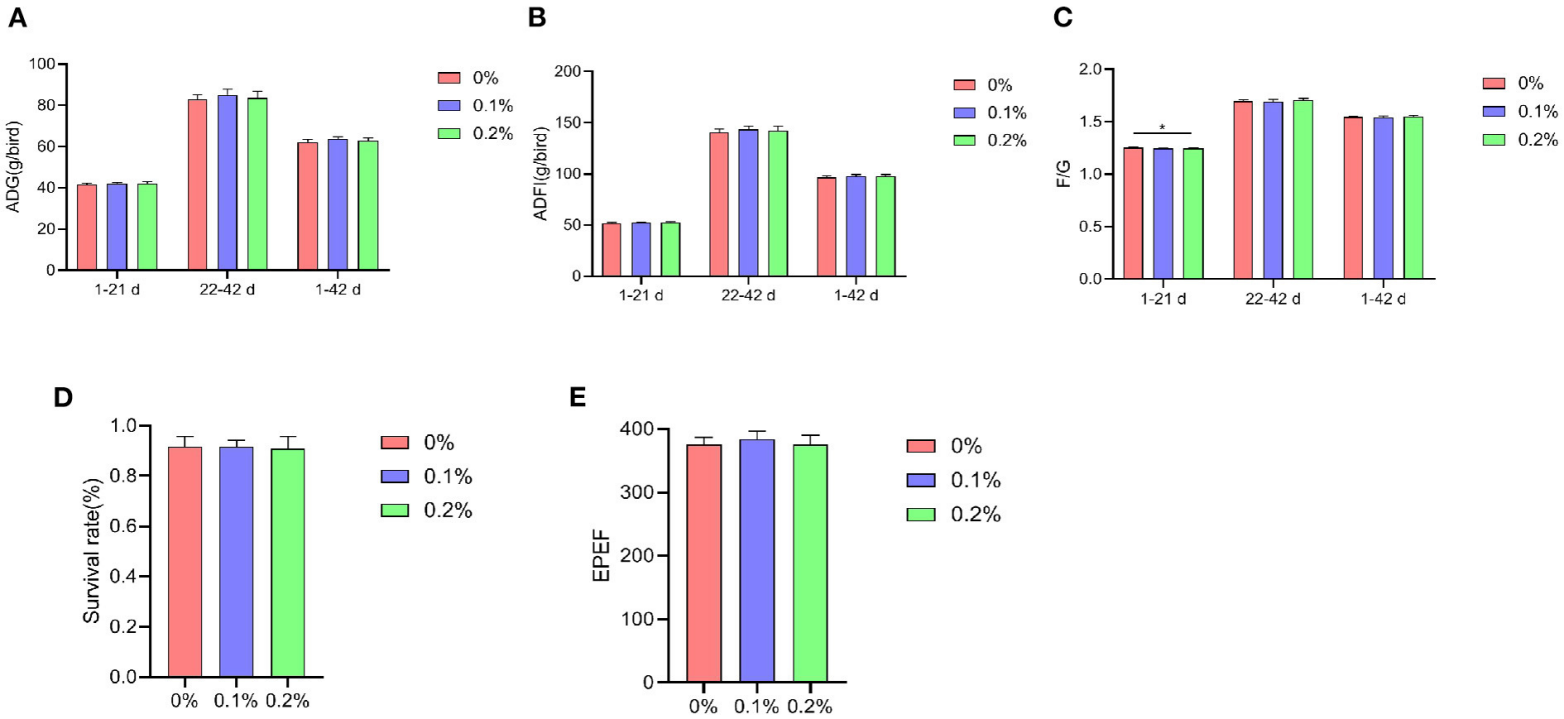
Effect of PC addition to diets on the growth performance in AA+ broilers. **(A)** Average daily gain. **(B)** Average daily feed intake. **(C)** Feed-to-weight ratio. **(D)** Survival rate. **(E)** European productivity efficiency factor. “*” means indicates a significant difference (*P* < 0.05). No “*” indicates that the difference is not significant (*P* > 0.05).

A shown in Figure 1, from 1–21 days, the F/G was significantly lower (*P* < 0.05) at a PC addition of 0.2% compared to the control group. Both ADG and ADFI improved to varying degrees in the test group, but did not reach significant levels (*P* > 0.05). At 22–42 and 1–42 days of age, ADG, ADFI and F/G were not significantly different between treatment groups (*P* > 0.05), while AA+ broilers had high values of ADG, ADFI and EPEF and low F/G when PC was added at 0.1%.

### Carcass traits and Immune organ index

**Figure 2:**
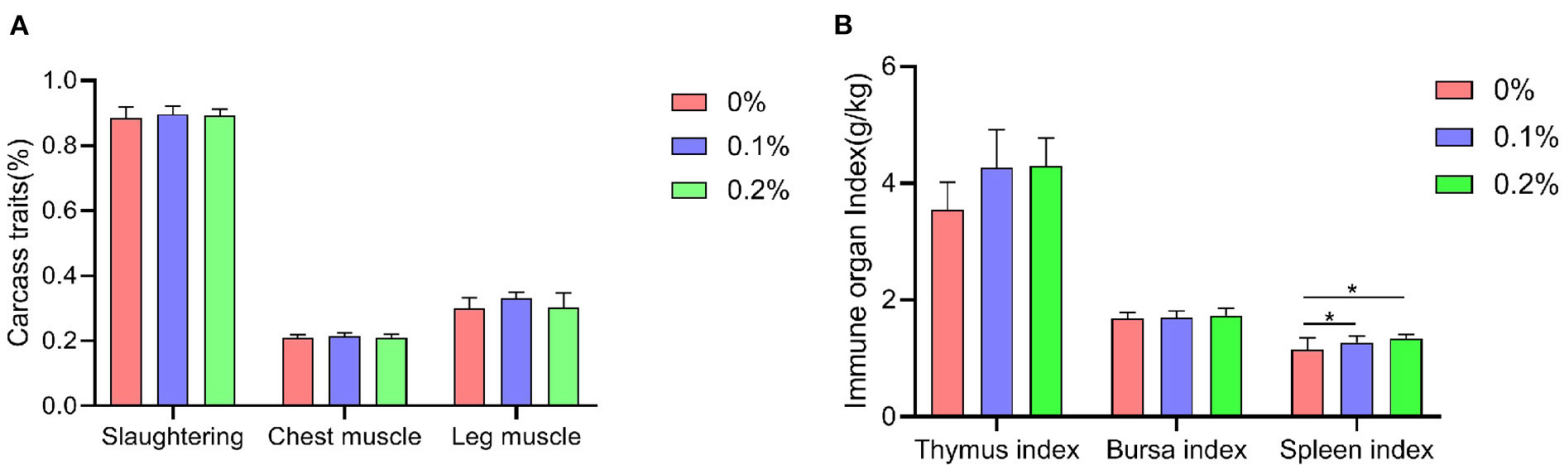
Effect of PC addition to diets on the carcass traits and immune organ index in AA+ broilers. **(A)** Carcass traits. **(B)** Immune organ index. “*” means indicates a significant difference (*P* < 0.05). No “*” indicates that the difference is not significant (*P* > 0.05).

A shown in Figure 2, dietary addition of PC had no significant effect (*P* > 0.05) on carcass traits in AA+ broilers, with high values for slaughter rate, breast muscle rate and leg muscle rate when PC was added at 0.1%. The addition of PC to the diet significantly increased spleen index in AA+ broilers compared to the control (*P* < 0.05), and increases in thymus and bursa index were also significant but did not reach significant levels (*P* > 0.05).

### Fecal microbiota

**Figure 3:**
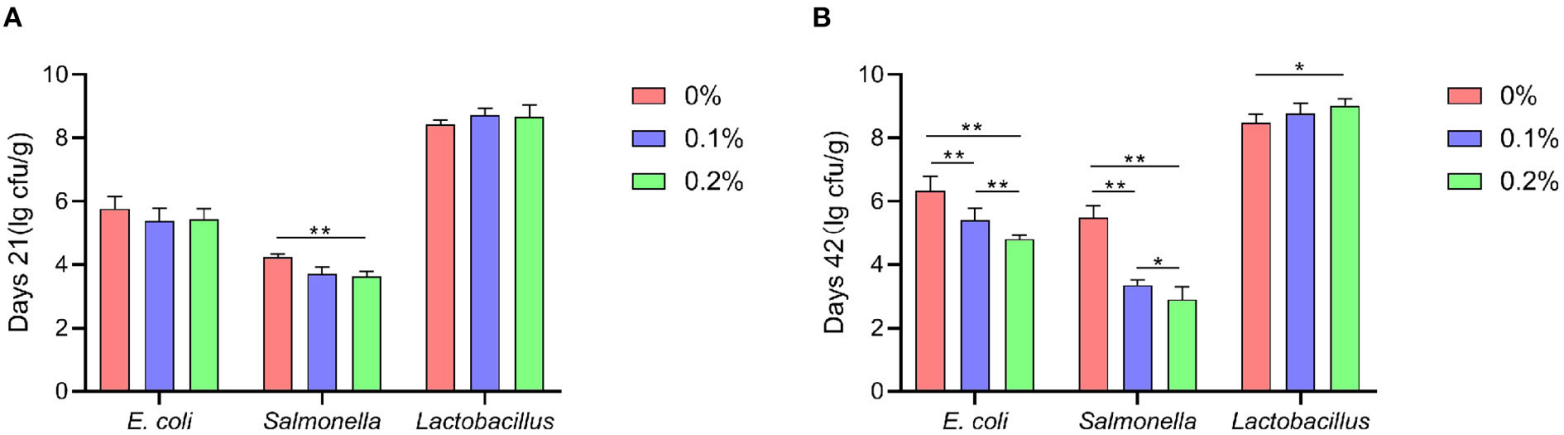
Effect of PC addition to diets on the fecal microbiota in AA+ broilers. **(A)** Days 21. **(B)** Days 42. “*” means indicates a significant difference (*P* < 0.05). “**” means indicates a extremely significant difference (*P* < 0.01). No “*” indicates that the difference is not significant (*P* > 0.05).

A shown in Figure 3, the addition of PC to the diet significantly reduced *Salmonella* levels in the feces of AA+ broilers at 21 and 42 days of age compared to the control group (*P* < 0.01). The *E. coli* content in the feces of AA+ broilers at 42 days of age was highly significantly reduced (*P* < 0.01). When PC was added at 0.2%, the content of *Lactobacillus* in the feces of AA+ broilers was highly significantly increased (*P* < 0.01).

### Fecal noxiousgas emissions

**Figure 4:**
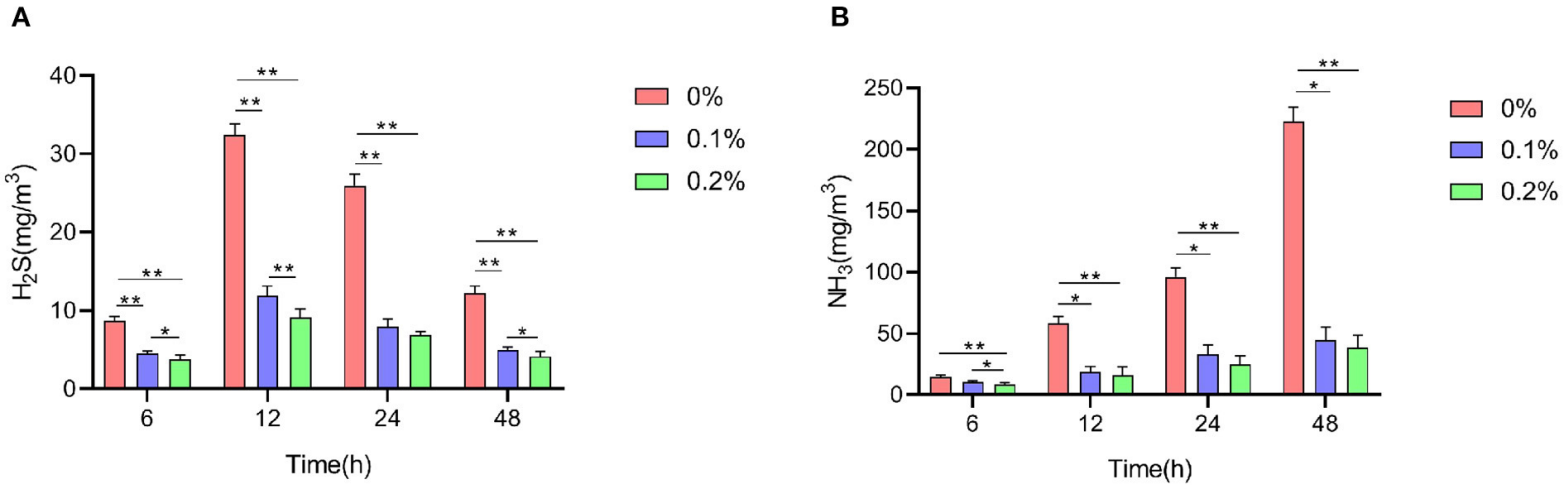
Effect of PC addition to diets on the noxious fecal gas emission in AA+ broilers. **(A)** H_2_S **(B)** NH_3_. “*” means indicates a significant difference (*P* < 0.05). “**” means indicates a extremely significant difference (*P* < 0.01). No “*” indicates that the difference is not significant (*P* > 0.05).

A shown in Figure 4, compared to the control group, the addition of PC to the diet significantly reduced the emission of NH_3_ and H_2_S from AA+ broiler manure at 6, 12, 24 and 48 h of fermentation (*P* < 0.01), and low values of NH_3_ and H_2_S emissions were observed when PC was added at 0.2%.

## Discussion

ADG, ADFI and F/G, as important indicators of production performance, had a direct impact on the economic efficiency of large-scale farms. EPEF, in turn, was used as an important indicator of broiler production, reflecting the performance of broilers, and as a profitability index, with a larger index indicating a more profitable chick (18). By adding a PC consisting of *Bacillus subtilis, Clostridium butyricum* and *Enterococcus faecalis* to the diet of AA+ broilers, it was found that F/G was significantly lower in the test group compared to the control group in the early growth period (1–21 d) and that the addition of 1% PC to the diet in the late growth period (22–42 d) improved the growth performance of AA+ broilers. Mountzoureis et al. (19) found that the addition of 0.1% of a complex probiotic to the diet had no effect on ADG and F/G in broilers at all stages. Gao et al. (8) found that when *Bacillus subtilis* was added at 200 mg/kg, the ADG of broilers was higher than that of the higher dose group with 250 mg/kg, and that the addition of *Bacillus subtilis* increased the ADFI of broilers but did not reach a significant level. Similar results were obtained in this trial, probably because we added too many probiotics to the AA+ broiler diet, which upset the balance of the animal's gut microbiota to the detriment of the animal's health. Zeng et al. (20) showed significant improvements in broiler growth performance following the addition of potential probiotics consisting of *Clostridium butyricum, Bacillus subtilis* and *Bacillus licheniformis* to AA+ broiler diets. The results of this trial also yielded similar results to those of the above researchers. When PC was added at 0.2%, there was a slight decrease in growth performance compared to the test group with 0.1%. This also suggests that probiotics should be used in moderation rather than in excess in broiler production.

Slaughter rate, breast muscle rate and leg muscle rate were crucial indicators of meat production performance in broilers and provided a useful assessment of carcass traits. The relative weights of the thymus, bursa and spleen serve as markers of organismal immunity in broilers, and the growth, development and division of immune cells could increase the weight of immune organs in broilers (21). The immune organ index, which was the ratio of immune organs to live body weight, provided some indication of the functional status of the animal's organism. The results of our trials showed that the addition of PC to the diet had no significant effect on carcass traits in AA+ broilers, with the highest slaughter rate, breast muscle rate and leg muscle rate being achieved when PC was added at 0.1%. Yadav et al. (22) reported that *Bacillus subtilis* supplementation to broiler diets had no significant effect on carcass traits, and Rehman et al. (23) similarly reported that the addition of probiotics did not affect carcass traits in broilers. Therefore, the highest slaughter, pectoral and leg muscle rates were seen in this trial when PC was added at 0.1%, probably because growth performance was highest when PC was added at 0.1%, resulting in higher carcass traits. The current study found that the addition of PC to the diet significantly increased the spleen index of AA+ broilers compared to the control group, and that the increase in thymus and bursa indices did not reach significant levels. This was also similar to the findings of Sjofjan et al. (24) and Gao et al. (25). The main reason might be that the beneficial flora in the probiotic complex multiply in the intestine and constantly synthesized vitamins, amino acids and other beneficial substances, which are indispensable for the growth and development of the animal's immune organs.

Air pollution in animal feeding environments had been widely recognized as a threat to animal health and safety, with ammonia and sulfur-containing compounds as two of the main noxious gases causing odor and environmental pollution problems on farms (26), and these contaminants could be detrimental to animal welfare. Han et al. (27) found that probiotics could indirectly reduce these environmental pollutants in animal feces by improving the intestinal micro-ecology. This pilot study found that dietary supplementation with a complex probiotic mixture of *Bacillus subtilis, Clostridium butyricum* and *Enterococcus faecalis* was effective in reducing the number of *E. coli* and *Salmonella* in feces, increasing the number of *Lactobacillus* and reducing NH_3_ and H_2_S emissions in feces. This was consistent with the findings of Zhang et al. (28) and Jeong et al. (29), as well as most researchers. Based on this test, it speculated that the addition of a probiotic complex maintains the intestinal microbial balance by increasing the number of beneficial bacteria in the gut and reducing the number of noxious bacteria, which in turn results in better growth performance and carcass traits.

## Conclusion

A probiotic complex made from *Bacillus subtilis, Clostridium butyricum* and *Enterococcus faecalis* can improve growth performance in AA+ male broilers, reduce fecal *E. coli* and *Salmonella* counts, increase *Lactobacillus* and reduce noxious NH_3_ and H_2_S emissions in feces.

## Data availability statement

The original contributions presented in the study are included in the article/supplementary material, further inquiries can be directed to the corresponding authors.

## Ethics statement

The animal study was reviewed and approved by the Animal Conservation and Utilization Committee of the JZMU. Written informed consent was obtained from the owners for the participation of their animals in this study.

## Author contributions

QZ: data curation, writing-original draft, and writing-review and editing. WM: investigation. TW: resources. XL: project administration, supervision, and validation. DL: resources, supervision, validation, writing, and writing-review and editing. All authors contributed to the article and approved the submitted version.
